# MOOCs and Medical Education: Hope or Hype? 

**DOI:** 10.15694/mep.2020.000124.1

**Published:** 2020-06-18

**Authors:** Ryan Clark, Leah Marks

**Affiliations:** 1 University of Glasgow

**Keywords:** MOOC, Blended learning, Futurelearn, Distance Education, Open Courses, Online Courses

## Abstract

This article was migrated. The article was marked as recommended.

The use of Massive Open Online Courses (MOOCs) in medical education is evolving and the potential of these learning and teaching resources has not yet been fully explored. Having previously integrated MOOCs within our medical undergraduate curriculum in a variety of contexts, in this study we focused on the attitudes of staff and students to the perceived utility of MOOCs in both undergraduate and postgraduate medical education. The results of an online survey demonstrate an openness to, yet in many cases, an unfamiliarity with, this technology in both educators and students. Further exploration of the data reveals a desire for greater levels of guidance in this ever expanding field, and specific curricular areas were highlighted where potential for the use of MOOCs seems particularly enticing. These insights may be helpful in the ongoing debate as to the direction of the field as educators seek to maximise the effectiveness of a blended approach to medical education.

## Introduction

Massive Online Open Courses (MOOCs) are internet-based courses, which are typically free to register for and can be accessed by large numbers of participants. The MOOC phenomenon has been mainstream in education for around seven years. In this time, it has diversified and matured in a variety of ways. MOOCs ‘for credit’ or for continuing professional development (CPD) have become commonplace as have programme MOOCs where learners study several MOOCs on related topics, receiving an award on completion ( Kiers and Van Der Werff, 2019). These developments have, controversially, highlighted the economic aspects of MOOCs: free access to the educational resources are often time-limited, with payment required for longer term access. Alongside this, developments in blending MOOCs into more traditional courses has also gained momentum in recent years ( Swinnerton *et al.,* 2017; Lambert and Altony, 2018).

At the University of Glasgow (UoG) we have developed our MOOC profile on the Futurelearn platform, showcasing some of the research and teaching being carried out in our institution. The School of Medicine has played a key role in this development, particularly with regards to introducing both blended learning and student co-creation of MOOCs into their elective curriculum ( Marks and Meek, 2018). Despite these innovative practices and local, small scale evaluations of individual MOOC initiatives, there remains a huge gap, in both literature and policy, as to the direction in which the field is moving. Some have suggested that, with the initial ‘hype’ over, MOOCs will gradually fade to become another underused and undervalued online tool (reviewed in Gul *et al.,* 2018). On the other hand, there is speculation that MOOCs could have an important role in both undergraduate learning and teaching as well as in lifelong learning and CPD ( Radford *et al.,* 2015; Bryson, 2017; Ibanez and Traxler, 2016; King *et al.,* 2018).

We are particularly interested in the potential use of MOOCs in undergraduate and postgraduate learning, specifically in a medical context. We believe MOOCs may have a role therein and, with continued pressure on doctors to embrace learning long after medical school, it seems likely that novel forms of CPD will be significant. Therefore, in this study we aimed to examine the current practice, experience and attitudes of staff and students in a Scottish Medical School to MOOCs, to help inform the direction of future developments.

## Methods

We found no validated tools for evaluation of perceptions and experiences which met the needs of our research. Therefore, with a view to using a mixed-methods approach for analysis, we developed two questionnaires (one for teaching staff and one for students) to investigate a range of parameters including previous experience of MOOCs and blended learning, as well as perspectives on future use. We carried out internal discussion to decide the themes that we wanted to address and developed these themes into groups of questions aimed which contained both Likert scale responses and space for free text comments (Supplementary file 1).

Using this questionnaire, we surveyed all teaching staff and undergraduate students at UoG via Online Surveys.com, to investigate their current understanding of MOOCs and probe how they felt these might be used in teaching and learning. After the initial email, reminders were sent out infrequently during the study period to encourage more responses. Analysis was performed through (1) tabulation of the quantitative results and (2) thematic textual analysis of the free-text comments. The study was approved by the University of Glasgow, MVLS Ethics Committee.

## Results/Analysis

### Staff Attitudes and Experience

Twenty-two members of staff responded to the survey, with nineteen being university-based educators and five clinical teachers ( Table 1). Ten knew how to access MOOCs, and nine had registered for one with seven participating and five completing. Only two educators currently used MOOCs in their own professional development while eight had considered it and none were against the concept. Fifteen expressed a desire for the university to recommend specific MOOCs. At the time of responding to the survey, two educators had incorporated MOOCs into their teaching with both finding this useful. Two said they would not be interested in using MOOCs created by others in their teaching with a further eight being ambivalent. Similar numbers were interested in using MOOCs created by themselves.

Eight members of staff felt the time required to create a MOOC was too great for the perceived reward, with only two knowing how to access funding for this. However, nobody disagreed with the statement ‘I would like to see more use of blended learning in Medical education’ with six remaining neutral. The majority (eleven) were neutral as to whether they would like to see more use of MOOCs in medical education with five and six disagreeing and agreeing respectively.

A key concern for some staff was whether the open nature of MOOCs made them unsuitable for medical education with six agreeing with this statement and five neutral. Videos and short articles were the components of MOOCs which were felt to be most useful. Overall no one disagreed with the statement that MOOCs have a place in medical education, although undergraduate rather than postgraduate education was seen as the most likely place. Useful areas for MOOCs were suggested as ‘basic areas of science teaching’ and the most commonly cited barriers were ‘students having the necessary time and motivation’, and the lack of ‘suitable’ or ‘quality’ material. Some comments also emphasized the critical nature of student interaction with staff, and clearly felt that the use of MOOCs could impede this.

**Table 1.** Summary of Staff and Student Responses

Staff		Students	
**Staff Participants**	22	**Student Participants**	40
**Familiar with term ‘blending’**	N/A	**Familiar with term ‘blending’**	13 (33%)
**Familiar with term ‘MOOC’**	N/A	**Familiar with term ‘MOOC’**	9 (23%)
**Have accessed a MOOC**	9 (41%)	**Have accessed a MOOC**	12 (30%
**Participated in MOOC**	7 (32%)	**Participated in** MOOC	7 (18%)
**Completed MOOC**	5 (23%)	**Completed** MOOC	5 (13%)
**Want more blended**	16 (73%)	**Want more blended**	17 (43%)
**Want more MOOCs**	6 (27%)	**Want more MOOCs**	19 (48%)
**Videos/articles/discussion**		**Videos/articles/discussion**	24/14/6
**Would like recommended**	15 (68%)	**Would like recommended**	28 (70%)
**Used in my teaching**	2 (9%)	**Used in my own education**	4 (10%)
**Would consider using in teaching**	12 (55%)	**Would consider using in teaching**	N/A
**Use in CPD**	2 (9%)	**Should be compulsory**	10 (25%)
**Have considered using in CPD**	8 (36%)	**Open nature means they are unsuitable**	3 (8%)

N/A = Not asked

### Student Attitudes and Experience

Forty students responded to the survey. Thirteen were aware of the meaning of a MOOC with nine being familiar with the concept of blended learning. Twelve knew how to access a MOOC, nine, seven and five had registered, taken part in and completed a MOOC respectively. Once the terms MOOC and blended learning were defined, four said they currently used MOOCs in their medical education while seventeen wanted more blending and nineteen wanted more use of MOOCs.

Recommendation of MOOCs was a key issue highlighted with twenty eight saying they would use them if specific MOOCs were recommended by the university although only ten felt they should be compulsory. Six students felt they took up too much time but only three felt the open nature of the resource made them unsuitable for use in a medical education context.

Short videos were favoured by twenty-four while discussions and articles by six and fourteen respectively. Only three disagreed with the idea that MOOCs had a role in medical education although this was perhaps slightly veering towards postgraduate education (with the ‘not certain’ response featuring highly). The range of potential areas of coverage was broad, from surface anatomy, microbiology to sleep medicine and ‘sensitive topics’ such as female genital mutilation. Factors which might limit their use were thought to be time, ease of access, preferred learning styles and quality of resources. There was some emphasis placed on the need for high quality face to face teaching and a leaning towards the use of MOOCs as a revision and consolidation tool rather than as the primary learning event. The results are summarised in Table 1.

## Discussion

The results of this survey show an openness to the use of MOOCs in medical education amongst both staff and students. Lack of exposure to this medium perhaps partly accounts for ambivalence in some responders although it must be taken into account that those choosing to participate may have a higher previous level of experience with the technology than their peers.

A reasonably high level of awareness and desire for the use of blended learning was noted in both groups, although the terminology does not have such widespread understanding as some might suppose. It was surprising that three quarters of students were unaware of what blended learning meant. However, it was encouraging to note that, when students were then asked about their opinions of blended learning using MOOCs, they responded positively. This interest students have in using online learning has been replicated in other fields: for example in one study on histopathology teaching, students were found to prefer online learning ( Herrmann *et al.,* 2015).

An interesting finding was both staff and student desire for courses to be ‘recommended’. This perhaps highlights an inherent uncertainty over what is useful, of high quality or appropriate. The theme of reassurance-seeking by medical students is not new but perhaps MOOCs could be used to develop students’ confidence in their own choice. Critical appraisal is a key skill in medicine and MOOCs have the potential to allow a better understanding of what makes sources useful.

Students also expressed concern that MOOCs would be compulsory, adding to an already large workload. However, rather than simply completing a course from start to finish, MOOCs are often ‘dipped into’, with learners choosing to engage with specific aspects of the courses ( Christensen *et al.,* 2013). This could also help students develop key skills in using information effectively - a GMC Outcomes for Graduates ( GMC, 2018).

It is also important to be mindful of accessibility and widening participation issues, currently high on the agenda of many medical schools. Even those students who are perceived as being IT literate may not be literate in all forms of educational technology. Furthermore, unequal access to both the technology required to successfully access these increasingly data hungry courses, and non-ubiquitous access to Wi-Fi, are issues which could make the implementation of these methods more difficult.

Clearly, there is an appetite for blended learning in medical education, at least at this one medical school. While medical schools operate in different socio-political and economic spaces, our work provides a starting point for the consideration of MOOCs as a viable blended learning method. MOOCs are more than simply hype: there is hope that they can provide high quality blended learning to future medical students and practitioners. How this develops in the future will depend on how this field expands as the perceptions defined in this study become better understood and acted upon.

## Conclusion

The use of MOOCs in medical education is, in many respects, still in its infancy. Promoting greater awareness of the enormous potential of these online educational tools may be useful as an initial step to harnessing their usefulness. However, coordinated and pedagogically focused strategies within and across medical schools will be necessary if this potential is to be fully realised.

## Take Home Messages

• MOOCs are a virtually untapped resource in medical education
• Surveying staff and students at our own institution revealed an openness to using MOOCS, alongside a reasonably high level of unfamiliarity with the technology

• Both staff and students indicated a desire for guidance, perhaps not unsurprising given the vast range of both subject material and quality within MOOCs
• Discussion within individual medical schools as to whether, and how, MOOCs may be used to support teaching and learning could be helpful

## Notes On Contributors

**Leah Marks** is a senior lecturer in Medical Genetics in the School of Medicine at the University of Glasgow (UoG). She has particular interest in the use of MOOCs in medical education. She was co-lead of UoGs first MOOC ‘Cancer in the 21st Century: The Genomic Revolution’ which has to date attracted over 55000 registrations and which has been ‘blended’ into the university’s medical Student selected components since 2015. ORCID: https://orcid.org/0000-0001-8037-9812

**Ryan Clark** is a final year medical student at UoG. He has work on and completed medical educational research in a variety of areas including: curriculum mapping, online learning, cross medical school comparisons of teaching and leadership and developing teaching skills for medical students. He is particularly interested in the use of new educational methods to enhance teaching of pathology.
