# Supplementary material for: MOOCs and Medical Education: Hope or Hype? 
Source: MedEdPublish (2016). 2020 Jun 18;9:124. [Version 1] doi: 10.15694/mep.2020.000124.1 (PMC10702632; doi:10.15694/mep.2020.000124.1)
Supplement: Peer review history

# Reviewer report: Reviewer response for version 1

**DOI:** 10.21956/mep.20021.r30552

**Published:** 2020-10-09

**Authors:** Barbara Jennings

**Affiliations:** 1 Norwich Medical School

This review has been migrated. The reviewer awarded 4 stars out of 5 Thank you to the authors for this interesting survey of staff and student perceptions and experiences of MOOCs within a medical school that has created its own MOOCs. In their introduction and discussion the authors make some excellent points about an appetite for blended learning and the potential for using MOOC content more widely in medical education and CPD – noting caveats about unequal access to digital resources and the need for signposting in the curriculum.In the methods and ethics statement the authors explain the recruitment of participants and data collection. The supplementary file presents the paperwork and questions used in the survey. I think the results section could have been more detailed, in particular regarding the thematic textual analysis and a detailed legend would have made table 1 easier to understand.I agree with the conclusion about the need to raise awareness of MOOC platforms and packages. In our medical school, I often discuss MOOCs in faculty and clinical supervisor development sessions; and have also noted that most colleagues are unfamiliar with MOOCs or don’t use them in practice. On the other hand, I think the colleagues who have developed MOOCs (also using FutureLearn platform) were well equipped to cope with the teaching adaptation that has been required in 2020 in response to the pandemic.I have to declare myself to be a MOOC cheer-leader and would love to see a multi-lingual MOOC developed to cover the basic and clinical sciences that are needed in all medical curricula; whether traditional or integrated or systems-based. I think high quality MOOCs could improve on most didactic face to face teaching and could be complemented by active small group learning and clinical placement. I was interested to see the finding that some students thought the open nature of the resource made them unsuitable for use in a medical education context. I wonder if the authors could elaborate on that point?

Reviewer Expertise:

NA

No decision status is available

# Reviewer report: Reviewer response for version 1

**DOI:** 10.21956/mep.20021.r30551

**Published:** 2020-06-25

**Authors:** Nandalal Gunaratne

**Affiliations:** 1 Faculty of Medicine, Wayamba University of Sri Lanka

This review has been migrated. The reviewer awarded 3 stars out of 5 A small number of participants but a good attempt to identify needs and usefullness. It is unfortunate that the word "Massive" is used to describe these as students are scared away by the term itself! I believe the faculty must be responsible in recommending a course after doing it themselves to fellow staff and to students.

Reviewer Expertise:

NA

No decision status is available

# Reviewer report: Reviewer response for version 1

**DOI:** 10.21956/mep.20021.r30553

**Published:** 2020-06-19

**Authors:** P Ravi Shankar

**Affiliations:** 1 American International Medical University

This review has been migrated. The reviewer awarded 4 stars out of 5 This interesting article studies faculty and student perception regarding MOOCs. The major benefit of MOOCs is access to world class resources and teachers to students in less privileged settings. I have completed a few MOOCs and participated in many more. The authors can share details of the questionnaire which they had used, though table 1 does contain some of the questions asked. It will be also interesting to know what percentage of faculty and students had participated in the survey. Among the limitations it is possible that those who had participated had greater experience with and a more positive attitude toward MOOCs. I would also be interested in knowing how MOOCs are blended with the course of study at the author’s institution. MOOCs can have a number of benefits in medical education and a process for grading the quality of MOOCs can be initiated.

Reviewer Expertise:

NA

No decision status is available
